# Arogenate dehydratases: unique roles in light-directed development during the seed-to-seedling transition in *Arabidopsis thaliana*

**DOI:** 10.3389/fpls.2023.1220732

**Published:** 2023-08-02

**Authors:** DurreShahwar Muhammad, Hussien F. Alameldin, Sookyung Oh, Beronda L. Montgomery, Katherine M. Warpeha

**Affiliations:** ^1^ Department of Biological Science, University of Illinois at Chicago, Chicago, IL, United States; ^2^ MSU-DOE Plant Research Lab, Plant Biology Laboratories, East Lansing, MI, United States; ^3^ Agricultural Genetic Engineering Research Institute (AGERI), Agriculture Research Center (ARC), Giza, Egypt; ^4^ Cell and Molecular Biology Program, Michigan State University, East Lansing, MI, United States; ^5^ Department of Biochemistry and Molecular Biology, Michigan State University, East Lansing, MI, United States; ^6^ Department of Microbiology and Molecular Genetics, Michigan State University, East Lansing, MI, United States; ^7^ Department of Biology, Grinnell College, Grinnell, IA, United States

**Keywords:** photomorphogenesis, hypocotyl, phytochrome, phenylalanine, seedling, hormone, seed-to-seedling transition

## Abstract

The seed-to-seedling transition is impacted by changes in nutrient availability and light profiles, but is still poorly understood. Phenylalanine affects early seedling development; thus, the roles of arogenate dehydratases (ADTs), which catalyze phenylalanine formation, were studied in germination and during the seed-to-seedling transition by exploring the impact of light conditions and specific hormone responses in *adt* mutants of *Arabidopsis thaliana*. *ADT* gene expression was assessed in distinct tissues and for light-quality dependence in seedlings for each of the six-member *ADT* gene family. Mutant *adt* seedlings were evaluated relative to wild type for germination, photomorphogenesis (blue, red, far red, white light, and dark conditions), anthocyanin accumulation, and plastid development-related phenotypes. ADT proteins are expressed in a light- and tissue-specific manner in transgenic seedlings. Among the analyzed *adt* mutants, *adt3*, *adt5*, and *adt6* exhibit significant defects in germination, hypocotyl elongation, and root development responses during the seed-to-seedling transition. Interestingly, *adt5* exhibits a light-dependent disruption in plastid development, similar to a *phyA* mutant. These data indicate interactions between photoreceptors, hormones, and regulation of phenylalanine pools in the process of seedling establishment. ADT5 and ADT6 may play important roles in coordinating hormone and light signals for normal early seedling development.

## 1 Introduction

Higher plant development from seed to seedling is an intricate multi-component process. This transition involves a switch from heterotrophic growth to light-driven autotrophy. Biologically active wavelengths of light, hormones, and nutrients act through an unknown number of signal transduction mechanisms to initiate autotrophic growth and coordinate many aspects of higher plant development (Borevitz et al., 2002; Sullivan and Deng, 2003; Warpeha and Montgomery, 2016). Light and hormones affect gene expression, where ~20% or more of the plant genome is regulated by white light (Jiao et al., 2005) and nearly 15% of the genome may respond to hormones (Nemhauser et al., 2006). Partitioning, regulation, and use of metabolic resources are still poorly understood, but postulated to be under the control of light and circadian regulators (Farré and Weise, 2012). What is known about photoreceptor-, hormone-, and metabolite-dependent regulation, and interactions among these factors during development from seed to seedling have been recently reviewed (Warpeha and Montgomery, 2016).

Among metabolites critical to the seed-to-seedling transition process is phenylalanine (Phe), which was recently reviewed (Perkowski and Warpeha, 2019). Phe is an amino acid utilized both in protein synthesis and as the precursor to thousands of compounds used by the plant (Hahlbrock and Scheel, 1989; Kliebenstein, 2004; Tzin and Galili, 2010; Maeda and Dudareva, 2012), including protectants against environmental signals, and that are directly upregulated by blue light (BL) and ultraviolet light (UV) (Bruns et al., 1986; Ohl et al., 1989). Phe can account for up to a third of the total organic carbon in a plant (Van Heerden et al., 1996), is the first committed precursor of the phenylpropanoid pathway, and is a concentration-limiting substrate for phenolics, phenylpropanoids, and other key compounds (Margna, 1977; Margna et al., 1989b; Maranville and Zhu, 2000; Rohde et al., 2004; Voll et al., 2004; Warpeha et al., 2008; Para et al., 2016). Arogenate dehydratase (ADT) enzyme isoforms are regulators of Phe in plants as they are involved in the final step of Phe biosynthesis (Fischer and Jensen, 1987). Cho et al. (2007) described six members of the *ADT* gene family, namely, *ADT1* (*At1g11790*), *ADT2* (*At3g07630*), *ADT3* (*At2g27820*), *ADT4* (*At3g44720*), *ADT5* (*At5g22630*), and *ADT6* (*At1g08250*), encoding proteins identified primarily in chloroplasts of light-grown plant material. *ADT3* (then called *prephenate dehydratase1* (*PD1*) (Warpeha et al., 2006) before the preferred substrate arogenate was indicated in detailed studies (Cho et al., 2007; Yamada et al., 2008) is expressed in young etiolated seedlings (Para et al., 2016), unlike other ADTs studied in more mature tissues. However, a recent study indicated that sequence conservation of amino acid residues conferring prephenate dehydratase activity is retained in plants throughout evolution (El-Azaz et al., 2016), and in vascular plants, relaxed feedback inhibition by Phe may have a profound impact on the induction and biosynthesis of phenylpropanoids (El-Azaz et al., 2022), raising many interesting possibilities for regulation of Phe.

Recent studies have revealed activities of ADT proteins that could impact seedling establishment. In 3- to 5-week-old *Nicotiana benthamiana* leaves with transiently expressed *A. thaliana ADT* genes, ADT2 localized to a chloroplast pole and at the chloroplast equatorial plane in a ring indicating involvement with chloroplast division machinery (Bross et al., 2017). ADT5 was identified in the nucleus, but the functional reason is unknown (Bross et al., 2017). In addition, Chen et al. reported that ADT proteins, and ADT2 the most, also contribute to anthocyanin accumulation (Chen et al., 2016). Recently, ADT2 was also shown to be vital for seed development (El-Azaz et al., 2018). *ADT3* is expressed at high levels in seed development (Rippert et al., 2009), and in young seedlings (Warpeha et al., 2006; Warpeha et al., 2008; Para et al., 2016). Phe supplied by ADT3 indicated roles in carbon storage, chloroplast development, cell division, and cell lineage commitment and cell morphology in the epidermis, and coordinates reactive oxygen species (ROS) homeostasis in young seedlings (Para et al., 2016).

It is not known how *ADT* expression affects germination processes or the transition to a photosynthetically competent seedling. Gene expression profiles at specific times have indicated that transcripts of *ADT2* and *ADT3* are expressed in seeds (Rippert et al., 2009), only *ADT3* in young (~4 days old) etiolated seedlings (Warpeha et al., 2006; Para et al., 2016), and in young seedlings in general (*ADT2* was only expressed in prolonged white light at 6 days) (Warpeha et al., 2006); other *ADT* genes are reported to be primarily expressed in older tissues (Rippert et al., 2009). Utilizing a number of genetic mutants, Corea et al. (2012) showed that some *ADT* genes regulate the carbon flux and then later lignin biosynthesis in maturing (3-week-old and older) plants.

The seed-to-seedling transition is a simple system with which to study abiotic signal responses, prior to full maturation of the photosynthetic apparatus. Light perceived by photoreceptors is central to germination, the transition from heterotrophy to autotrophy, hypocotyl elongation, and cotyledon and leaf development during seedling establishment (Franklin and Quail, 2010; Kami et al., 2010). Hormones also contribute to the seed-to-seedling progression. Abscisic acid (ABA), gibberellins (GAs), auxins (e.g., indole-3-acetic acid [IAA]), and ethylene are among the hormones that all contribute to this process (reviewed in Warpeha and Montgomery, 2016). ABA and, to a lesser degree, auxins prevent early germination, whereas GA and ethylene promote germination. GA and auxins promote hypocotyl elongation. Light and hormone signaling pathways interface during the seed-to-seedling transition, and this is still not well understood (Warpeha and Montgomery, 2016).

The seed-to-seedling transition is a rapid process where a program of embryonic dormancy is phased out in favor of an independent vegetative organism, which includes the transition of the cotyledons from storage to photosynthesizing organs in Arabidopsis that includes chloroplast development. Some nutrients and other compounds are stored in the seed, but the network of metabolic induction and control in seedling development is still a complicated puzzle. To better understand potential Phe dynamics, we explored potential roles of individual ADT family members during the seed-to-seedling transition.

## 2 Materials and methods

### 2.1 Plant materials

Columbia WT and T-DNA insertions originally obtained from ABRC/TAIR were utilized (Alonso et al., 2003). Null mutant insertions for *Pirin1* (*PRN1*; At3g59220; SALK_006939) were utilized as controls in some experiments, and T-DNA insertion mutant line *sig6-1* (AT2G36990; SAIL_893_C09) was used in the RNA-seq experiment. Null ADT mutants were tested in genetic and developmental assays, including *ADT1* (At1g11790; SALK_138343; SALK_124232), *ADT3* (At2g027820; SALK_029949; SALK_071907), *ADT4* (At3g44720; SALK_065483; SALK_123367), *ADT5* (At5g22630; SALK_088171; SALK_028611), and *ADT6* (At1g08250; SALK_030329; SALK_109552). All mutant phenotype responses were confirmed in two accessions; the more common null mutant utilized in research (accession listed first) was featured in the data shown. Bulk seed stocks were grown as previously reported (Orozco-Nunnelly et al., 2014). T-DNA insertion *phyA* and *phyB* mutants used in these studies were previously isolated and described (Mayfield et al., 2007; Ruckle et al., 2007). The *phyAphyB* mutant was obtained from a genetic cross of the single mutants as described (Oh and Montgomery, 2013). Seed lines completely null for *ADT2* (At3g07630) have not been available. Recently, it has been proven that ADT2 null mutation is lethal because ADT2 is essential for proper seed development (El-Azaz et al., 2018), and hence, *ADT2* is not studied herein.

### 2.2 Light-dependent hypocotyl and root elongation phenotyping assays

Seeds were surface sterilized and plated on 1X MS containing 1% sucrose and 0.7% Phytoblend (Caisson Laboratories, UT) essentially as previously described (Warnasooriya and Montgomery, 2009). Plated, sterilized seeds were cold stratified during imbibition in darkness at 4°C for 4 days. All experiments were conducted at constant temperature and humidity under continuous light in controlled-environment chambers. Light sources were those previously described (Warnasooriya and Montgomery, 2009) with continuous red, R (Rc; λ_max_ ~ 670 nm), continuous far-red, FR (FRc; λ_max_ ~ 735 nm), and continuous blue, B (Bc; λ_max_ ~ 470 nm) at the indicated fluences in µmol m^−2^ s^−1^. Continuous darkness is signified by Dc or 7D0L.

For light-dependent hypocotyl elongation experiments, hypocotyl lengths of seedlings grown under defined light conditions were measured by scanning the seedling images and quantifying lengths using ImageJ software (Plug-in used to stitch pictures that required two images to get whole seedling). We measured 25 seedlings in each of three independent biological replicates, resulting in 75 measured seedlings per line. For vertical plate assays, hypocotyl and root lengths were compared to a scanned ruler image taken at the same time by scanning and photography on day 7, post-stratification. After hypocotyl and root lengths were measured, the mean ratio of hypocotyl length:root length was determined.

### 2.3 Vertical growth phenotyping and germination assays

Seeds were surface sterilized and plated on 0.5X MS (pH 5.8) with no sucrose and no added vitamins as described (Voll et al., 2004) on square (positioned vertically) petri plates or phytatrays, as detailed herein. For germination assays on phytatrays, sterilized seeds were mixed with 0.8% low melt agarose (0.5X MS; top agarose) with the addition of 1 μM ACC or 500 nM ABA or 1 μM IAA or 1 μM GA or 0.5X MS (control); then, a 100-μl aliquot of seed and top agarose was spotted onto a 0.5X MS phytatray base plate, where germination assays were performed similarly to Kim et al. (2019). Plated, sterilized phytatrays were placed into light-tight black Plexiglas boxes, sealed with aluminum foil, and then were cold stratified in a dark cold room at 4°C for 48 h (Kim et al., 2019). After 48 h, the plates were moved to one of several locations: to a 24-h, 20°C dark environmental room, or a white light environmental room (Orozco-Nunnelly et al., 2014) for varying numbers of days, ranging from 0 to 7 days, depending on the experiment. White light sources, as well as dim green light for handling dark-grown seedlings, have been described (Voll et al., 2004); red and blue Plexiglas and filters used for supporting (low fluence) work have been described (Warpeha et al., 1989), where the fluence rate received by the vertical plates determined by a LiCor meter was 3 (Rc) and 7 (Bc) μmol m^−2^ s^−1^, respectively. All accessions of mutants were tested in these experiments; one representative accession is featured in the figures.

### 2.4 Constructs and transformation of *adt* mutants

*ADT* construct development and subsequent plant transformation occurred as has been described (Orozco-Nunnelly et al., 2014). Standard molecular biology techniques and the Gateway system (Invitrogen) were used in the cloning procedures of the *ADT* family members described herein. Purified WT Arabidopsis cDNA was used to generate the *ADT* full ORF fragments for each *ADT* member (except *ADT2* as aforementioned), where each ORF was represented by *ADT1* [1,179 bp], *ADT4* [1,275 bp], *ADT5* [1,278 bp], and *ADT6* [1,242 bp], and where *ADT3* cloning of the ORF was described previously (Para et al., 2016). Each *ADT* ORF fragment was cloned into the Invitrogen pENTR/D-TOPO vector. LR reactions (Invitrogen) were then performed with verified (i.e., by sequencing in both directions) entry clones to obtain expression clones. The *35S* promoter was cloned as described (Orozco-Nunnelly et al., 2014), and the dpGreen binary vector derivative containing a NOS terminator with a C-terminal GFP fusion and spectinomycin and BASTA resistance genes was used, resulting in each *ADT* construct (*35S*::*ADTX*-*GFP*), except for *ADT3*, which was cloned for an earlier study as described with the native promoter (Para et al., 2016), and separate construct utilizing the 35S promoter. All constructs were confirmed via restriction enzyme digest, PCR, and sequencing. Verified expression clones were transformed in *adt* mutant backgrounds via floral dip as described (Para et al., 2016). *35S::ADTX-GFP* was introduced into null mutants (*ADT1* [At1g11790; SALK_138343]; *ADT3* [At2g27820; SALK_029949]; *ADT4* [At3g44720; SALK_065483]*; ADT5* [At5g22630; SALK_088171]; and *ADT6* (At1g08250; SALK_030329]) via the floral dip method as described, and screened by BASTA resistance in each generation until homozygous (T3) for experimentation (Orozco-Nunnelly et al., 2014). For each *ADT*, the four most vigorous T3 lines were selected for experiments. Living seedlings were mounted in sterile water on slides at the same time (~9 a.m.) in the day cycle, then viewed on a Zeiss Observer.Z1 deconvoluting microscope fitted with a high-resolution camera (Axiovision 503; Zeiss, Oberkochen, Germany), using the 20× objective to view expression fluorescence, sectioned by optical apotome (1-μm optical slices, no bleed through) illuminated by XCite 120 LED (Lumen Dynamics, Waltham, MA USA) DAPI, FITC, and Texas Red LEDs. Images were managed by Zeiss Zen pro software (2012). At least 30 seedlings were viewed per experimental replicate, with three biological replicates performed. Merged images of DAPI, FITC, and Texas Red LEDs are shown.

### 2.5 Germination and phenotyping

Germination was scored as complete emergence of the radicle as described (Kim et al., 2019). Phenotypic responses on untreated (control) and experimental hormone plates were determined by comparison of mutants to Columbia WT on a Zeiss Stereo Discovery V.8 microscope at 1× using Axiovision with images recorded, and entire plates were photographed on day 4 after stratification using Nikon Coolpix on a white light box or black background. At least three sets of 30 seeds were scored for germination on phytatrays at 0 to 72 h every 4 h, post-stratification.

### 2.6 Anthocyanin extraction and quantification

Anthocyanins were extracted and quantified from 5-day-old seedlings as previously detailed (Montgomery et al., 1999; Warnasooriya and Montgomery, 2009). At least 100 seedlings per line from four biological replicates were used.

### 2.7 Heat map analysis

Heat maps were constructed using AtGenExpress public Arabidopsis microarray datasets with mean-normalized values (www.weigelworld.org ) and visualization tools from BAR Heatmapper Plus (http://bar.utoronto.ca ). For tissue-specific data, seedlings were 7 days old grown in continuous white light (Wc). Light-pulsed seedlings were 4 days old.

### 2.8 Far-red block of greening treatment

The far-red block of greening (FR-BOG) experiment was performed according to Alameldin et al. (2020); Arabidopsis seeds were surface sterilized with 35% (v/v) commercial bleach containing 0.025% (v/v) SDS for 15 min. Seeds were rinsed five times with sterile distilled water and planted on growth medium containing 0.5X MS salts (Caisson Laboratories, Smithfield, Utah, USA) and 0.7% (w/v) Phytoblend (Caisson Laboratories). Seeds on solid medium were stratified for 4 days at 4°C in the dark and were then divided into two groups. One group was incubated in a Percival LED (light-emitting diode)-equipped growth chamber (model E-30LED; Percival, Perry, IA, USA) at 22°C under constant far-red LED (FRc; λ_max_ ~ 735 nm) light at 5 μmol m^−2^ s^−1^ for 5 days, then in a Percival environmental chamber model no. CU36LA under Wc at 100 μmol m^−2^ s^−1^ for 5 days. The second group was kept in the dark (D) for 5 days at 22°C as the control treatment, then grown under 100 μmol m^−2^ s^−1^ Wc for 5 days.

### 2.9 Chlorophyll assay

Ten-day-old seedlings were soaked in 200 µl of N,N-dimethylformamide per milligram fresh mass and kept in the dark for 24 h at 4°C (Moran, 1982). The absorbance of samples was then measured at 647 nm, 664 nm, and 900 nm (baseline control) using an Agilent 8453E UV-visible spectrophotometer (Santa Clara, CA, USA). Chlorophyll content was calculated based on previously described equations (Inskeep and Bloom, 1985). All assays were done with at least three biological replicates.

### 2.10 RNA library preparation and sequencing

Total RNA was extracted from whole seedlings (FR BOG-treated and controls) grown at 22°C using the OMEGA E.Z.N.A Plant RNA kit (catalog no. R6827). cDNA was synthesized using a Reverse Transcription System (Quanta bio) qScript cDNA SuperMix (Omega Bio-tek, Norcross, GA, USA) and the instructions of the manufacturer. Libraries were prepared using the Illumina Stranded mRNA Library Kit and Ligation with IDT for Illumina RNA UD Indexes following the manufacturer’s recommendations except that half volume reactions were used. Completed libraries were quality checked and quantified using a combination of Qubit dsDNA HS and Agilent 4200 TapeStation HS DNA1000 assays. All libraries were normalized down to the lowest concentration and equal volumes of these normalized libraries were pooled. The pool was quantified using the Invitrogen Collibri Quantification qPCR kit. The library pool was loaded onto one lane of an Illumina NovaSeq 6000 S4 flow cell and sequencing was performed in a 2 × 150-bp paired-end format using a NovaSeq v1.5 300 cycle reagent cartridge. Base calling was done by Illumina Real Time Analysis (RTA) v3.4.4, and output of RTA was demultiplexed and converted to FastQ format with Illumina Bcl2fastq v2.20.0.

### 2.11 RNA-Seq data analyses

An initial quality check of the RNA reads was performed using FastQC (https://www.bioinformatics.babraham.ac.uk/projects/fastqc/ ). Trimmomatic v0.32 (Bolger et al., 2014) was used to filter the RNA reads to remove adaptors and low-quality reads. A sliding window method was used to scan the reads with 4-base wide and cut when the base quality was below a threshold of 2. The minimum read length cutoff was 100 bp. Data quality was explored after filtering with FastQC. STAR/2.6.0c (Spliced Transcripts Alignment to a Reference; Dobin et al., 2013) was used to map the RNAseq reads to the TAIR10.1 *Arabidopsis thaliana* genome (RefSeq assembly accession: GCF_000001735.4) with the default settings of the twopassMode Basic option with intron size 21–6,000 nt. In all samples, >90% of the RNAseq reads were mapped to the reference genome. RNA‐Seq data have been deposited to the NCBI Gene Expression Omnibus database (BioProject accession number: PRJNA991474).

### 2.12 Differential expression and clustering analyses

The HTseq-count function in HTseq (High-Throughput sequencing) v0.6.1 (Anders et al., 2015) was used in the default mode and stranded=yes for generating read counts. HTseq-count output was fed into DESeq2 (Love et al., 2017) for differential expression analysis using the standard steps represented in the DESeq function (Love et al., 2014). A gene was considered differentially expressed if the adjusted *p*-value <0.05 and the |log fold change| > 1 and had a Transcript Per Million (TPM) > 1 in at least one condition. The *p*-value was adjusted with a *q*-value false discovery rate (Benjamini and Hochberg, 1995).

### 2.13 Statistical analyses

There were different kinds of data collected, including hypocotyl measurements, numbers of germinated seeds, pigment content, and expression data. Appropriate statistical tests were selected based on the experiment. Germination data were plotted in GraphPad Prism showing mean and error based on SD with error bars indicated. Data were considered significant when *p* was <0.05. For chlorophyll content and light-dependent hypocotyl elongation experiments in R, FR, and B light, data were analyzed using one-way ANOVA, with the *post-hoc* Tukey HSD test applied where relevant (https://astatsa.com/OneWay_Anova_with_TukeyHSD/ ). For anthocyanin content, two-tailed, unpaired Mann–Whitney *U*-test analyses were performed to compare the means of anthocyanin contents per milligram of fresh weight for a particular mutant line relative to WT in either darkness or light. For the 0- to 7-day seedlings grown in darkness (7D 0L), white light (0D 7L), or 3D4L, and the low-fluence 3-day seedlings (**Supplementary Figures 2, 3**), at least three replicates of 10–20 seedlings each were analyzed where the unpaired *t*-test was used; for some samples, applying Welch’s correction was appropriate (GraphPad Prism, v.9). Statistical analyses used for differential gene expression and clustering analyses are described in section 2.12.

## 3 Results

### 3.1 Expression of *ADT* genes is tissue-specific and light-regulated in *Arabidopsis*

**Figure 1:**
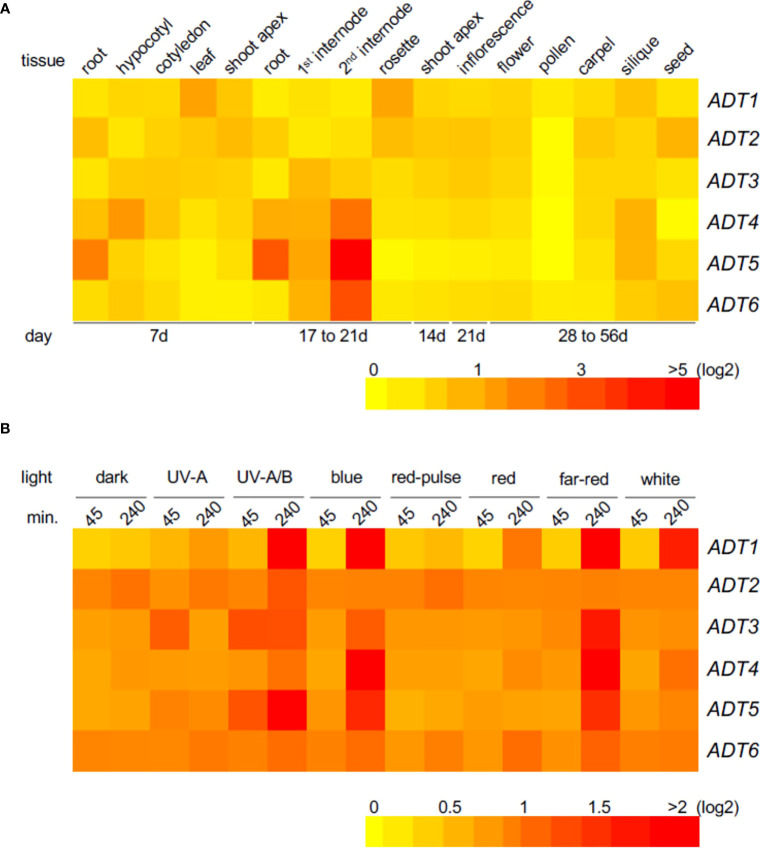
Heat map showing the expression of *ADT* genes in various Arabidopsis tissues **(A)** or under different light conditions **(B)**. Mean-normalized values from AtGenExpress expression library (www.weigelworld.org ) and BAR Heatmapper *Plus* (http://bar.utoronto.ca) were used for heat map construction. For tissue-specific expression **(A)**, 7-day seedlings were grown in continuous white light (far left) and other ages of seedlings shown under the map **(B)**. Four-day-old seedlings grown on MS medium were treated with specific light for either 45 or 240 min and aerial parts (hypocotyl and cotyledons) were used to extract RNA. Color scale represents log2 expression values; red indicates high expression and yellow denotes low expression.

To obtain insight into tissue localization and regulation of expression of the *ADT* genes, we constructed heat maps using Arabidopsis AtGenExpress public microarray datasets of young Co-0 WT seedlings (www.weigelworld.org ; **Figure 1**). *ADT* genes are expressed at relatively low levels in most tissues, and in a tissue-specific manner in 7-day seedlings grown in Wc (**Figure 1A**). Previously reported data using qRT-PCR analyses (Rippert et al., 2009) indicated some similarities, and some major differences with these data, but age and growth conditions including light levels were distinct for the respective information. Given the prior association of some *ADT* genes with light regulation (Warpeha et al., 2006), we assessed the impact of distinct light qualities in 4-day-old seedlings. Each ADT member indicated responsiveness to all light qualities (**Figure 1B**). Notably, *ADT* genes were expressed at higher levels with a longer irradiation, the exception being *ADT2*, which expresses at higher levels regardless of light condition, with the exception of UV-A, UV-A/B, and R pulse where a longer irradiation (or in darkness, a longer treatment) increased induction. Most *ADTs* expressed at higher levels in longer irradiations of UV-A/B, B, and FR.

*In planta* tissue localization of fluorescently tagged ADT proteins was localized by exploring each tissue of the seedlings by microscopy. Green fluorescence above background indicated expression comparable to the transcript analysis results (**Figure 1A**) for 7-day plants. Each *ADT* gene family member GFP reporter construct (35S promoter, except for *ADT3* that utilized the native promoter due to a larger study (Para et al., 2016) was transformed into a corresponding null *adt* mutant. Seedlings were grown under basta selection, under a long-day (16-h light:8-h dark; 16:8) growth condition, then live seedlings were viewed at day 7 post-planting. ADT protein, which may accumulate distinctly based on protein stability and/or turnover relative to 35S promoter-driven gene expression, was observed by microscopy as generally low throughout seedlings, with specific, predominating tissue-specific patterns for each ADT member (**Supplementary Figure S1**). ADT1-GFP and ADT6-GFP accumulated in cotyledon mesophyll cells, particularly in the chloroplasts (confirmed by fluorescence pattern [21]), but where ADT1 was sporadic throughout the mesophyll (from individual chloroplasts to many chloroplasts), ADT6 was detected in most mesophyll cells in all sections through the cotyledon. Although ADT3-GFP exhibited low expression in newly divided cells and SAM, it was most discernable in the cotyledon epidermal layer, especially at the plasma membrane of pavement cells and in immature guard cells, which confirms reported results for young dark-grown seedlings (Para et al., 2016). ADT4-GFP was most expressed in the upper hypocotyl, occurring largely in plastids. ADT5-GFP was the only ADT with a distinct expression in the root. Null *adt2* is embryonic lethal (El-Azaz et al., 2018), so ADT2 was not studied in this context.

### 3.2 Germination phenotypes of *adt* mutants

**Figure 2:**
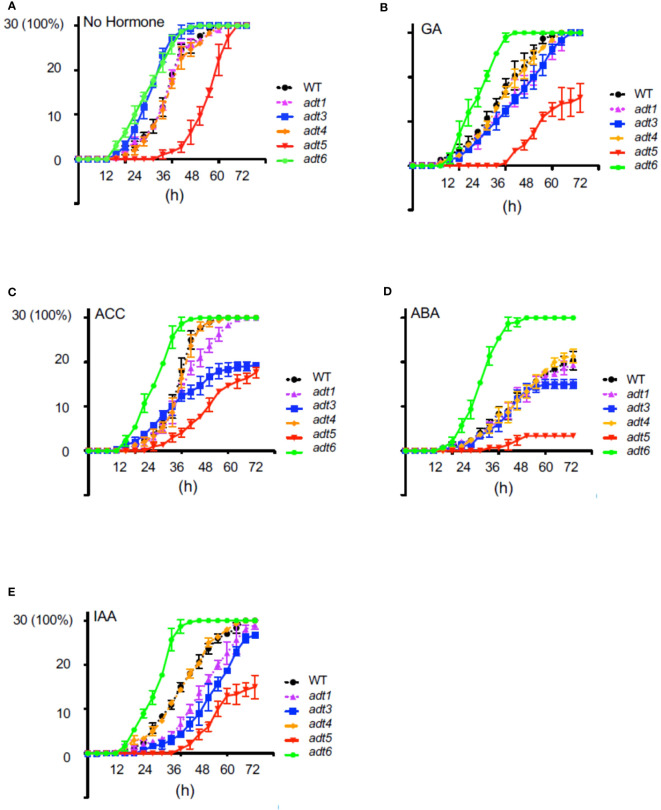
Germination of wild-type and *adt* mutants in the absence or presence of hormones. WT or *adt* mutant seeds were grown as described in Methods inclusive of exogenous hormones, shown in the figure. Thirty seeds (*y* axis; 30 seeds germinated = 100%) were sown with **(A)** no exogenous hormone, i.e., No Hormone (Control); **(B)** gibberellic acid (GA) at 1 μM; **(C)** 1-aminocyclopropane-1carboxylic acid (ACC) at 1 μM; **(D)** abscisic acid (ABA) at 500 nm; or **(E)** indole-3-acetic acid (IAA) at 1 μM. Germination was scored every 4 h up to 72 h where resultant data were plotted in GraphPad Prism indicating mean and SD as shown.

To elucidate the role of ADT-mediated Phe synthesis during the earliest stage of the seed-to-seedling transition, we investigated the role of ADT in the seed germination process. Given the recognized role of hormones in modulating the timing of seed germination, seeds of *adt* mutants were grown in complete darkness in the absence or presence of a specific hormone in the medium (**Figure 2**). All *adt* seeds germinated similarly to WT (~100%) by 72 h post-stratification in the absence of exogenous hormone (No Hormone [NH]; **Figure 2A**). However, *adt5* exhibited a delay in germination compared to WT, whereas *adt6* and *adt3* germinated before WT by 24 h. *adt1* and *adt4* demonstrated germination patterns similar to WT. GA at 1 μM had unique effects on the germination of the *adt* mutants (**Figure 2B**). *adt6* seeds germinated more quickly than WT. *adt4* germinated similarly to WT, whereas *adt3* and *adt1* germination was delayed but within range of WT responses. Germination of all *adt* mutants in the presence of GA was similar to WT (~100%) by 72 h, except *adt5*, which indicated delay in any germination up to ~36 h, then gradual germination increase over the 72-h period. In response to ACC, *adt6* germinated rapidly within 24 h, similar to the NH condition (**Figure 2C**). *adt1, adt3*, and *adt4* germination were delayed by growth on ACC similarly to WT, with approximately 50% of seeds germinating by 36 h, but between 36 and 72 h, *adt1* germination continued in a linear fashion, behind WT and *adt4*, with 100% germination by 72 h. *adt3* seed germination decreased from 36 to 72 h with just over 60% fully germinating. *adt5* like NH indicated a slow to start germination process, but then exhibited linear germination over time, with only ~60% germinated by 72 h. ABA at 500 nM caused an expected delay in WT relative to untreated (NH) seeds, with, *adt1*, *adt3*, and *adt4* indicating similar delays, where *adt3* did not fully recover after 48 h (**Figure 2D**). ABA blocked *adt5* germination, even by 72 h, post-stratification. *adt6* appeared completely insensitive to ABA, germinating at the same rate as NH. In response to IAA, WT was slightly delayed, and *adt1*, *adt3*, and *adt5* were inhibited in increasing effect (i.e., *adt5* was the most inhibited, where the overall profile resembled responses to GA and ACC) (**Figure 2E**). Considering germination overall with or without hormones, *adt4* had near-identical germination responses as WT. *adt6* was uniquely unresponsive to all hormones, and *adt5* was uniquely delayed in germination in response to all hormones, where ABA appeared to completely shut down germination.

### 3.3 Role of ADT in regulating far-red block of greening and ABA signaling

**Figure 3:**
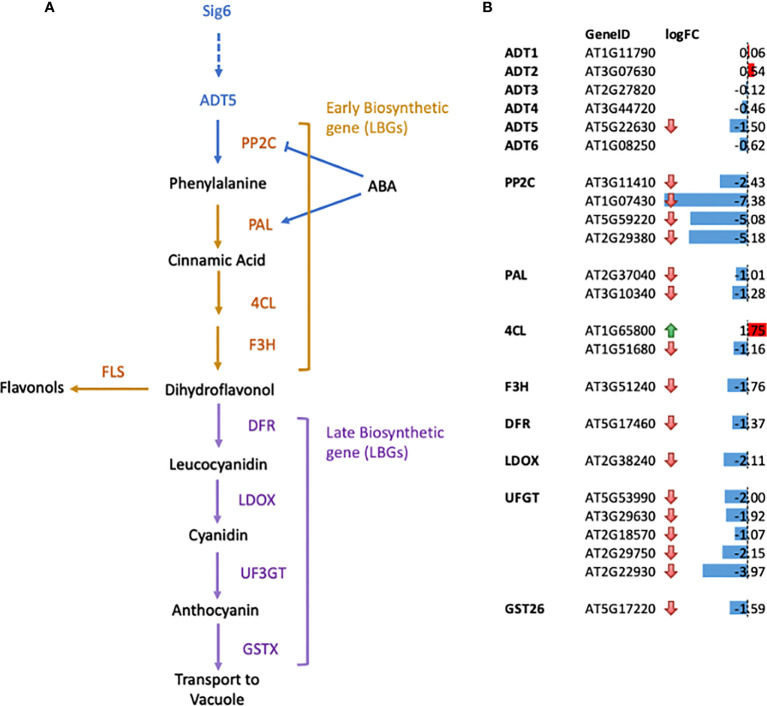
Gene expression changes for genes in the flavonoid biosynthetic pathways in *sig6* relative to wild-type under far-red block-of-greening (FR-BOG) conditions. **(A)** Flavonoid biosynthetic pathway. Orange and purple letters indicate the early biosynthetic genes (EBGs) and the late biosynthetic genes (LBGs), respectively. **(B)** Expression of genes involved in flavonoid biosynthesis in *sig6* under FR-BOG. Values are average obtained from four independent RNAseq data experiments. PP2C, protein phosphatase 2C; PAL, phenylalanine ammonia-lyase; 4CL, 4-Coumarate : CoA ligase; F3H, flavonol 3-hydroxylase; F3′H, flavonol 3′-hydroxylase; FLS, flavonol synthase; DFR, dihydroflavonol-4-reductase; LDOX, leucoanthocyanidin dioxygenase; UF3GT, UDP-glucose flavonoid-3-Oglucosyltransferase; GST26, Glutathione S-transferase 26.

Given the impact of ABA on *adt5* germination and that ABA impacts transcription of chloroplast genes by a PP2C-dependent activation of nuclear encoded sigma factors (SIGs) (Woodson et al., 2013; Yamburenko et al., 2015), we examined interplay between ADT family members and plastid development. As phyA is known to regulate sigma factors, especially Sig6 (Oh and Montgomery, 2013), and they are both involved in the plastid development-related FR-BOG response in plants (Alameldin et al., 2020), we assessed FR-BOG-dependent gene expression in a *sig6* mutant (**Figure 3**).

**Figure 4:**
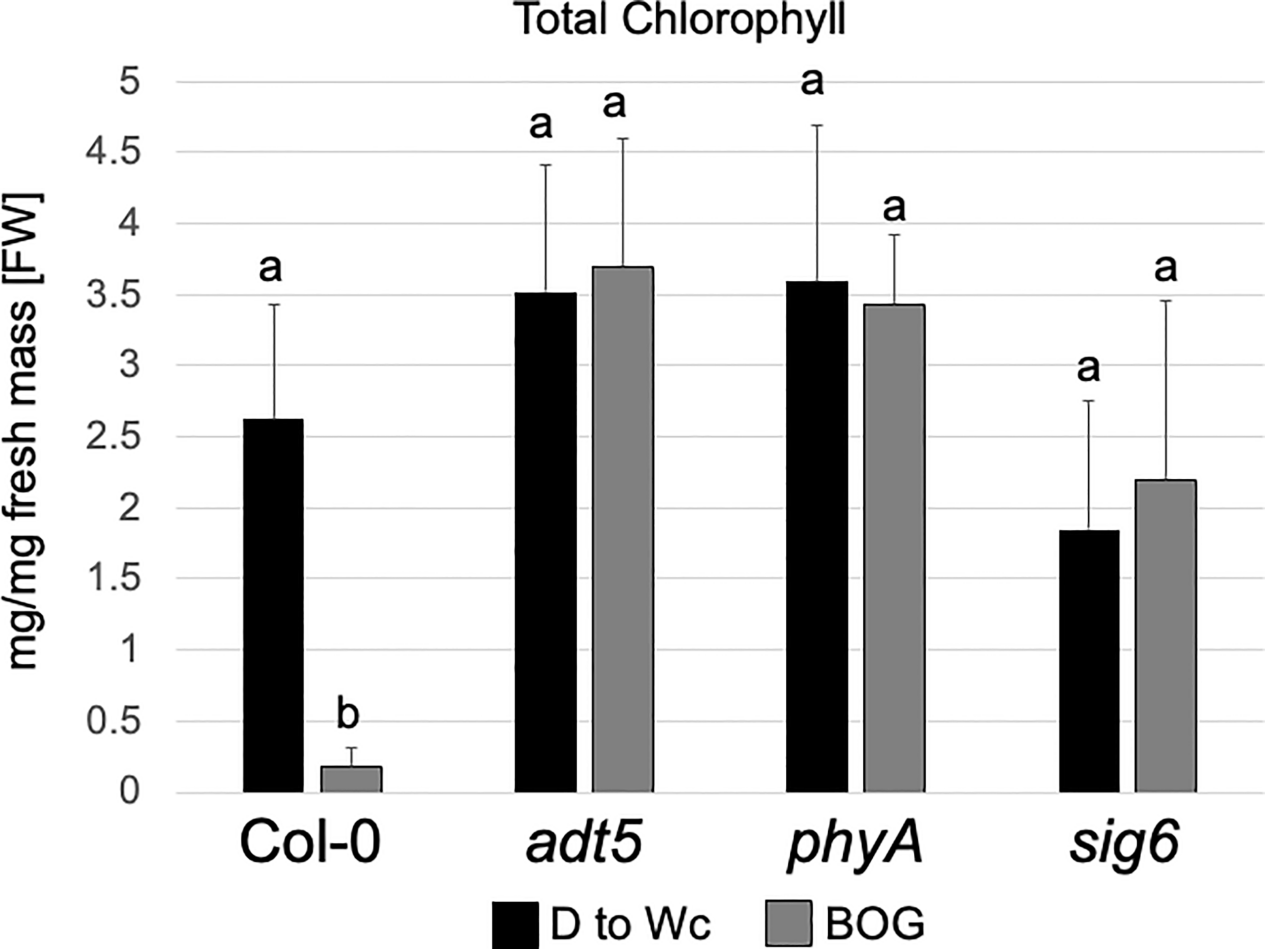
Chlorophyll content of wild-type, *adt5*, *phyA*, and *sig6* lines. Chlorophyll content of Col-0 WT, *adt5*, *phyA*, and *sig6* mutant lines was grown at 22°C in darkness (D) for 5 days, then moved to continuous white light (Wc) at 100 μmol m^−2^ s^−1^ for 5 days, i.e., control treatment (D to Wc), or under constant far-red (FRc) illumination at 5 μmol m^−2^ s^−1^ for 5 days, then moved to Wc at 100 μmol m^−2^ s^−1^ for 5 days, i.e., FR block-of-greening (BOG) treatment. Total chlorophyll (mg/mg fresh mass [FW]) was extracted with N,N-dimethylformamide. Data are presented as means ( ± SD) and were analyzed using a one-way ANOVA, which revealed a significant main effect of genotype (*F*_7, 110 =_ 9.09, *p* < 0.0001), a significant effect of treatment (*F*_1, 110 =_ 301.48, *p* < 0.0001), and a significant interaction between factors (*F*_7, 110 =_ 14.46, *p* < 0.0001). Bars marked with different letters are significantly different (*p* < 0.05) based on the Tukey–Kramer *post-hoc* test using astatsa.com.

Our RNA-seq data of the *sig6* mutant line under FR-BOG conditions indicated that *ADT5* was the only ADT gene that was downregulated in the *sig6* mutant line under FR-BOG treatment compared to growth in white light (**Figure 3B**), suggesting a potential role of ADT5 in the FR-BOG response. To examine phenotypes that may be predicted by the alteration of *ADT5* mRNA levels in a *sig6* mutant under FR-BOG compared to wild type, we tested the greening response of the *adt5* line under FR-BOG. Our results indicated that, like *phyA* and *sig6* that have been previously shown to have an altered greening response in FR-BOG (Alameldin et al., 2020), the *adt5* mutant similarly accumulates chlorophyll under FR-BOG (**Figure 4**). This result supports a potential role of ADT5 in the chloroplast development process.

To further understand the role of ADT5 in the ABA-dependent chloroplast development process, we investigated ABA-related genes in our RNA-seq dataset. Our data indicate the misregulation of numerous ABA response genes (**Figure 3B**). Multiple members of the PP2C protein family, known to regulate ABA signaling negatively, are downregulated in *sig6* under FR-BOG conditions [56, 57. 58].

We detected a downregulation of two phenylalanine ammonia-lyase (PAL) genes in a *sig6* mutant under FR-BOG (**Figure 3**); PAL catalyzes the conversion of phenylalanine to trans-cinnamic acid, the first step of phenylpropanoid biosynthesis leading to diverse plant metabolites reactions (Truman et al., 2007; Widjaja et al., 2009). PAL is also involved in the ABA signaling pathway, where PAL was misregulated in *aba3* mutants under oxidative stress. Our data also indicated the downregulation of the Cinnamate 4-hydroxylase *4CL1* gene that controls the last step of the general phenylpropanoid pathway (Li et al., 2015).

Additionally, we detected a downregulation of flavanone 3-hydroxylase (F3H) that is coordinately expressed with chalcone synthase and chalcone isomerases and is involved in flavonoid biosynthesis (Shih et al., 2008). Flavonol synthase 3 (FLS3), involved in the last step of flavonol biosynthesis (Pelletier et al., 1999), was also downregulated in the *sig6* mutant under FR-BOG. Furthermore, five of the UDP-Glycosyltransferase superfamily members’ mRNA levels were significantly reduced in the *sig6* mutant under FR-BOG. UDP-Glycosyltransferase superfamily proteins play a central role in the last step of the anthocyanin biosynthesis pathway (Yonekura-Sakakibara et al., 2014). Additionally, the GLUTATHIONE S-TRANSFERASE PHI 12 (GSTF12), which functions as a carrier to transport anthocyanin from the cytosol to tonoplasts (Wagner et al., 2002), was also downregulated in the *sig6* mutant under FR-BOG. Since *adt5* was delayed in germination in response to ABA, and given the fact that ADT5 regulates phenylalanine biosynthesis, our RNA-seq data analysis suggests that ADT5 might play an important role in the anthocyanin biosynthesis pathway.

### 3.4 *adt* mutant seedlings have unique responses to darkness and white light

After emergence of the seedling from the seed, and prior to emergence into light (presumably under soil or leaf litter in natural contexts), seedling development initiates with rapid stem elongation in darkness. Following emergence into a light environment, photoreceptors perceive ambient light, then initiate the inhibition of stem elongation and promotion of cotyledon expansion, plastid development, and root development. Until the seedling is fully autotrophic, these processes are supported by metabolites stored in the seed, and made in the emerging seedling. We investigated the role of ADTs in darkness, continuous light, or as a result of light-to-dark-transition, from 0 to 7 days.

#### 3.4.1 Seedling development in complete darkness

**Figure 5:**
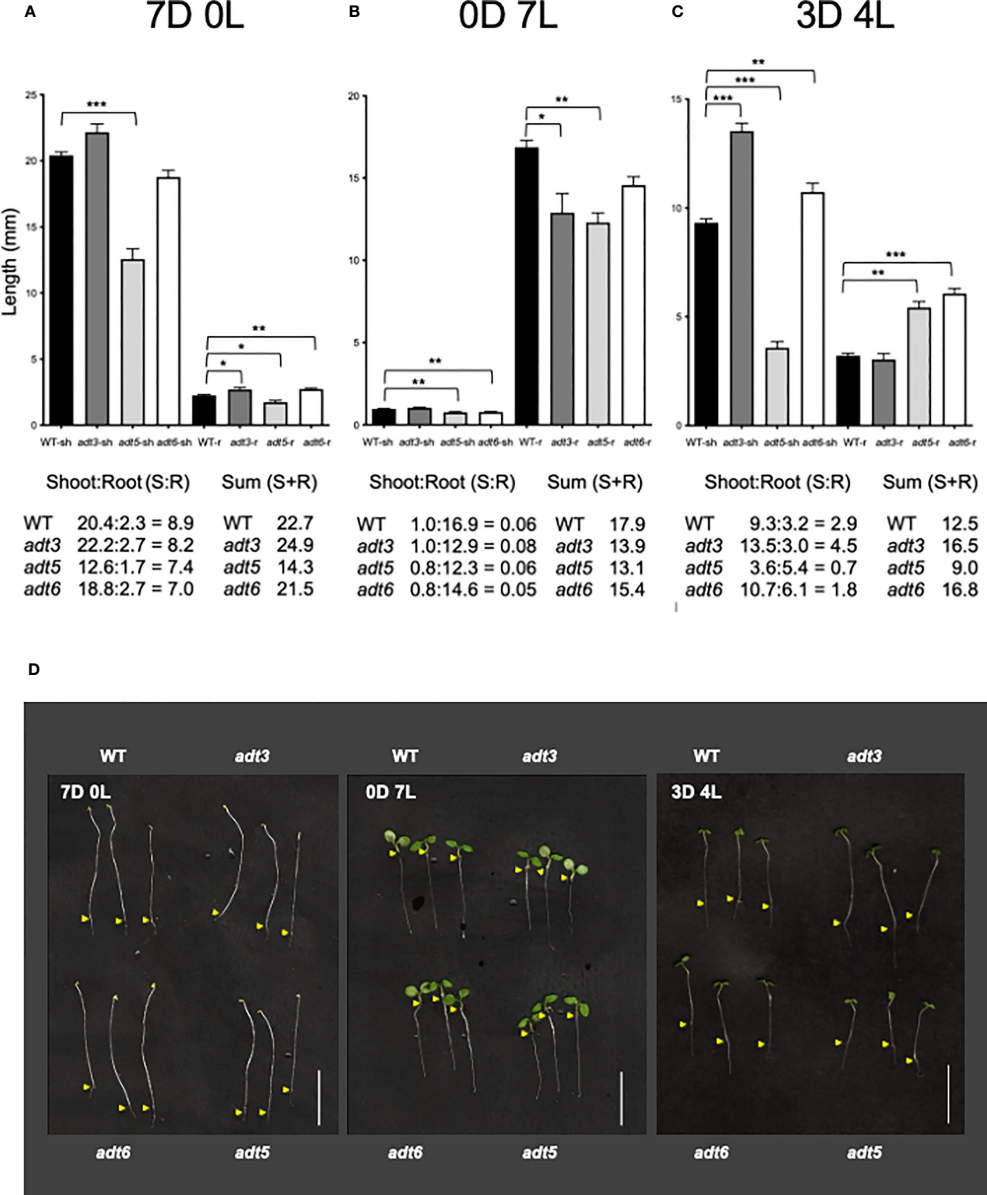
Darkness and white light 7-day growth assays. WT and *adt* seedlings were grown vertically on sucrose-free 0.5XMS plates under conditions of **(A)** continuous darkness (7D0L), **(B)** continuous white light (0D7L), or **(C)** a dark–light transition (3 days darkness; 4 days light, 3D4L), maintained at 20°CC. Lengths of shoots (sh) and roots (r) were measured, then bar graphs were made in GraphPad Prism, with differences in mean lengths of roots and shoots compared by unpaired *t*-test, as reported in Results. Lengths of sh and r were summed (shoot + root) in mm. Ratios of shoot:root were also calculated as shown. **(D)** Representative seedlings are shown in the images; scale bar for images indicates 1.0 cm. Yellow arrows indicate the root–shoot junction. *p < 0.05, **p < 0.01., ***p < 0.001.

The *adt1* and *adt4* mutants were not significantly different from WT in shoot or root development in the dark or any light condition tested (**Supplementary Figure S2**); hence, the remainder of the light study focused on *adt3*, *adt5*, and *adt6* mutants. When grown in Dc (7D0L), hypocotyl (shoot) elongation (shorter; *p* < 0.001) and root elongation (shorter; *p* < 0.05) were significantly different for the *adt5* mutant compared to WT (7D0L; **Figure 5A**). *adt6* mutants grown in Dc also exhibited shorter shoots relative to WT, but the difference was not significant (*p* = 0.083), whereas roots were significantly longer than WT (*p* < 0.01), ultimately resulting in similar shoot+root total lengths for *adt6* and WT (**Figure 5A**). *adt3* mutant seedlings grew longer in the Dc compared to WT, in both shoot (n.s., *p* = 0.2665) and root (*p* < 0.05) lengths, resulting in an overall increased total shoot+root mean length. Although the summed total length of shoot and root were similar for *adt6* and less for *adt5* relative to WT, both *adt5* and *adt6* exhibited reduced shoot:root ratios. Shoot:root ratios can be impacted by environmental conditions and can indicate relative energy contributions to shoot vs. stem elongation (Wilson, 1988).

#### 3.4.2 Seedling development in continuous white light

In continuous white light (0D7L), all roots elongated less than WT [*adt3* (*p* = 0.0138), *adt5* (*p* < 0.001), and *adt6* (n.s., *p* = 0.07) (**Figure 5B**)]. *adt5* and *adt6* shoot lengths were both less than that observed for WT (*p* < 0.01 for *adt5* and *adt6*). The ratios of shoot:root lengths for *adt5* and *adt6* were similar to WT in this light regime. *adt3* shoot length was similar to WT (n.s., *p* = 0.2766), considering that root length resulted in a shoot:root ratio larger than the WT ratio. The sums of the shoot+root length for all *adt* mutant seedlings were less than WT.

#### 3.4.3 Seedling development during a dark-to-light transition

The differences in 7D0L vs. 0D7L were distinct for *adt3, adt5*, and *adt6* mutant seedlings, and led us to test dark-to-light transitions. We tested periods of darkness (D) and periods of white light (L) from 6 days of Dc followed by moving seedlings to 1 day of white light (i.e., 6D:1L), then 5D:2L and so on to 1D:6L. We found that only the regime of 3D:4L resulted in any significant differences between *adt* mutants and WT seedlings (3D4L; **Figure 5C**). *adt3* exhibited a significantly longer shoot (*p* < 0.001), but only a marginally shorter root (n.s. *p* = 0.4796), producing a larger shoot:root ratio and larger shoot+root length compared to WT. *adt5* shoot elongation was less than that measured for WT seedlings (*p* < 0.001). *adt5* root elongation, however, was greater than WT (*p* < 0.01), with the shoot:root ratio and total summed shoot+root length of *adt5* being less than those of WT (**Figure 5C**). Conversely, *adt6* shoot length was longer than WT (*p* < 0.01), and the root length nearly double that of WT (*p* < 0.001). This phenotype resulted in a shoot+root length for *adt6* that exceeded WT, whereas the shoot:root ratio was less than WT.

In all three of the light regimes tested, *adt5* had reduced shoot+root lengths. *adt6* in contrast exhibited shoot+root length less than WT in Dc (7D0L) and Wc (0D7L), but greater than WT in the 3D4L condition. If there was darkness in the growth regime (7D0L and 3D4L), *adt3* exhibited greater shoot+root lengths. Representative images of the seedlings in the light regimes including controls are shown in **Figure 5D**. Given that *adt5* differs from WT in all conditions, whereas *adt3* and *adt6* differ from WT and each other in specific conditions, these data strongly suggest unique light-dependent roles for these ADT family members.

### 3.5 Monochromatic light-dependent and -independent impacts on hypocotyl elongation in *adt* mutants

**Figure 6:**
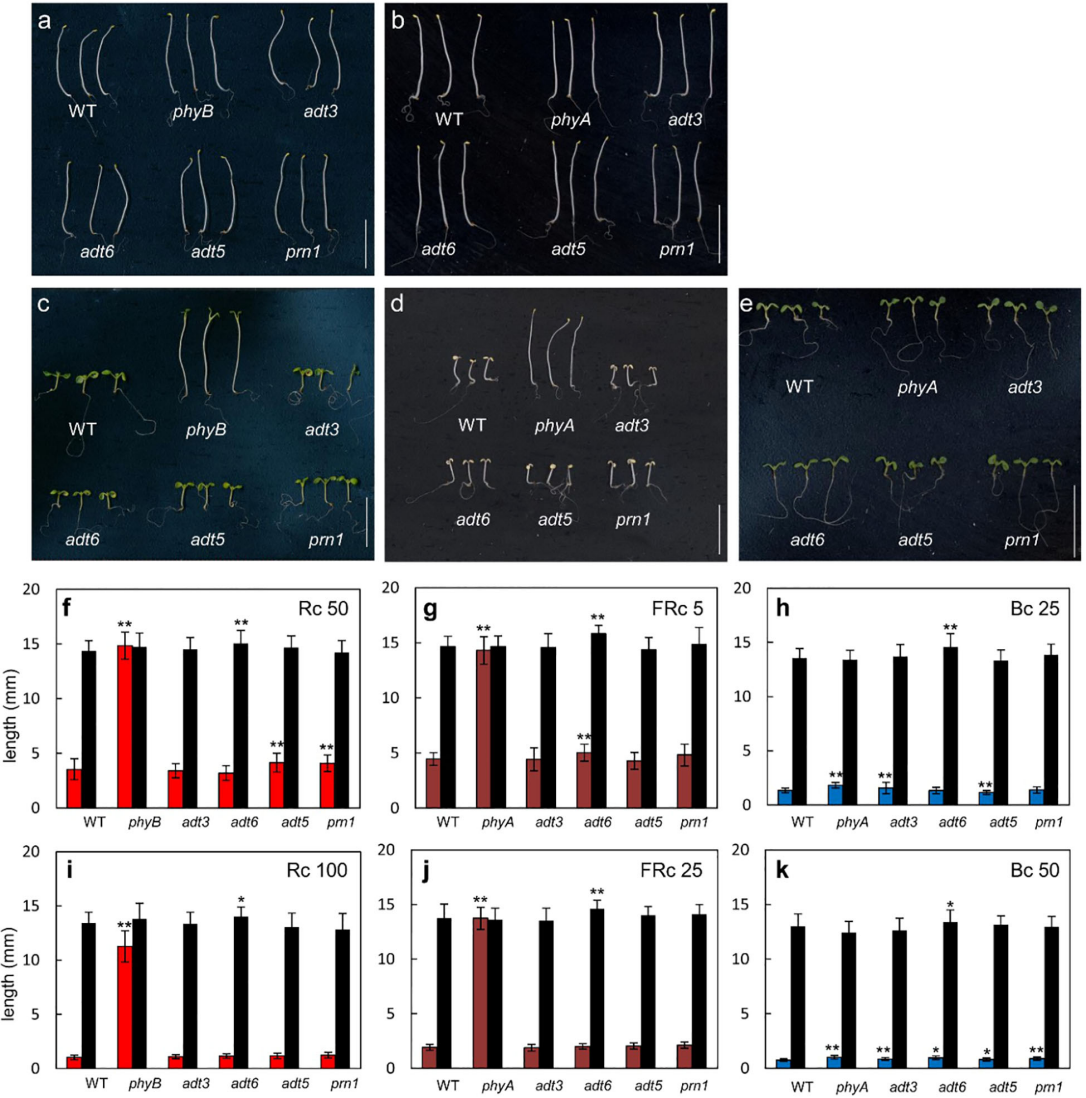
Photomorphogenesis of wild-type and *adt* seedlings. WT, *adt*, and *prn1* (control) lines were grown at 22°CC on Phytablend medium containing 1% sucrose for 7 days in **(A, B)** darkness, **(C)** continuous red light (Rc) of 50 μmol m**^−^** ^2^ s**^−^** ^1^, **(D)** continuous far-red light (FRc) of 5 μmol m^−2^ s^−1^, or **(E)** continuous blue light (Bc) of 25 μmol m^−2^ s^−1^. *phyB* mutant seedlings are included as control in Rc measurements and *phyA* mutant seedlings in FRc and Bc measurements. Hypocotyl length measurements of WT, *adt*, and *prn1* seedlings under **(F, I)** Rc, **(G, J)** FRc, and **(H, K)** Bc at the indicated fluence rate in μmol m^−2^ s^−1^; data points represent mean ( ± SD) of hypocotyl lengths measured for 25 seedlings of each line in each of three independent experiments, i.e., *n* = 75. Black bars are hypocotyl lengths in dark-grown seedlings. Data analyzed by one-way ANOVA with *post-hoc* Tukey’s test; *p < 0.05, **p < 0.01. Scale bar = 1 cm.

Owing to the responses of *adt3*, *adt5*, and *adt6* seedlings in variable light conditions, we measured hypocotyl lengths of these mutants in specific R, FR, or B monochromatic light conditions. Plants were grown at two different fluence rates of continuous R (Rc; 50 and 100 μmol m**^−^** ^2^ s**^−^** ^1^), FR (FRc; 5 and 25 μmol m**^−^** ^2^ s**^−^** ^1^), or B (Bc; 25 and 50 μmol m**^−^** ^2^ s**^−^** ^1^) light with a control set grown in Dc (black bars) for each light condition. A *phyA* mutant was used as a positive control for FR and B light, and a *phyB* mutant was used as a positive control for R light experiments. Of note, *adt3* has been previously shown to have a B-dependent role in Phe synthesis (Warpeha et al., 2006; Warpeha et al., 2008). An insertion mutant of Pirin1, *prn1*, was also included as it is involved in accumulation of phenylpropanoids, and in B- and ABA hormone-dependent signaling (Orozco-Nunnelly et al., 2014). These data are shown in **Figure 6** (representative seedlings for conditions are shown **Figures 6A–E**).

Under an Rc illumination of 50 μmol m**^−^** ^2^ s**^−^** ^1^, *adt5* and *prn1* were longer (*p* < 0.01 for both) than WT, similar to the *phyB* mutant (*p* < 0.01), though not to the same magnitude (**Figures 6C, F**). At a higher fluence of Rc (i.e., 100 μmol m**^−^** ^2^ s**^−^** ^1^), no *adt* mutants were significantly longer than WT (**Figure 6I**), suggesting a light intensity-dependent role. Under an FRc of 5 μmol m**^−^** ^2^ s**^−^** ^1^, the *adt6* (*p* < 0.01) mutants were longer than WT, which was similar but to a much lesser degree than the FR-light insensitive *phyA* mutant (*p* < 0.01; **Figures 6D, G**). Similar to Rc data, a higher fluence of FRc light did not indicate any significant differences of *adt* mutants and WT (**Figure 6J**). Under Bc at 25 μmol m**^−^** ^2^ s**^−^** ^1^, *adt3* was longer than WT (*p* < 0.01), similar to the positive control *phyA* (*p* < 0.01; **Figures 6E, H**). *adt5* was shorter than WT (*p* < 0.01) under these conditions (**Figures 6E, H**). Markedly, at 50 μmol m**^−^** ^2^ s**^−^** ^1^ of Bc, all of the mutants tested were significantly longer than WT (**Figure 6K**), similar to *phyA*. Notably, among the lines tested, *adt6* exhibited a consistently longer hypocotyl than WT in complete darkness on sucrose-containing medium (**Figure 6**). These results confirm the input of multiple wavelengths of lights into ADT function during seedling development, as predicted from the gene expression data (**Figure 1**). As Rc and Bc illumination impacted *adt5* and *adt6* development in hypocotyl inhibition assays on sucrose-containing medium at 7 days, we also examined seedlings during the early post-germination period under a lower fluence rate of Rc or Bc on sucrose-free medium, assessed at 3 days (72 h) post-stratification (**Supplementary Figure S3**). Shoots and roots of *adt6* were longer than WT under Rc and Bc, and achieved significance for all measurements (*p* < 0.001); *adt5* was significant for all measurements (Shoots, Bc *p* < 0.001; Rc *p* < 0.01) except Rc roots (n.s.).

### 3.6 Anthocyanin accumulation is impacted in *adt5* mutants

The accumulation of anthocyanins is promoted by light, and anthocyanins are synthesized from the Phe-dependent phenylpropanoid pathway (Chen et al., 2016). Thus, we assessed anthocyanin accumulation in specific single *adt* mutants grown under Wc conditions at the early seedling stage. Anthocyanin levels in the control *phyAphyB* mutant were reduced to 63% of WT levels (**Supplementary Figure S4**), and the *prn1* mutant that overproduces specific pigments in response to light (Orozco-Nunnelly et al., 2014) was also used as a control. Notably, the *adt5* mutant accumulated only ~70% of WT anthocyanin levels (**Supplementary Figure S4**). No other mutant had a light-dependent defect in anthocyanin accumulation under our conditions, though *adt5* and *prn1* accumulated less anthocyanins in Dc than that observed for WT (**Supplementary Figure S4**).

## 4 Discussion

### 4.1 ADT5 and ADT6 regulate early developmental responses

There are two aspects of the phenotypes of *adt* mutants that appear particularly important to consider—the germination or early phenotype, where germination is typically completed for WT by 48–72 h post-stratification, and the seedling phenotype, observed herein at 4–7 days. During germination (0–3 days), the available pool of resources, including Phe, stored in the seed to support early growth appears critical, prior to assembly and function of the chloroplasts, where expression of ADTs is deemed important as reviewed (Perkowski and Warpeha, 2019). During seedling development, ADT5 and ADT6 have different actions, which may be based on the tissue in which they primarily occur. As observed in the analysis of germination (**Figure 2**), it is clear that *adt5* mutants have difficulty in germination, which may partly be explained in that ADT5 is expressed in the roots (**Figure 1**; **Supplementary S1**) where roots emerge first from the seed. *adt5* seeds may possess insufficient resources in the seed to support germination. The inability of *adt5* mutants to overcome dormancy in the dark in response to germination-promoting hormones tested (ACC and GA) provides additional evidence of dependence on photoreceptors, perhaps reinforced by the fact that long irradiations of UV-A/B, blue, and far-red light increase the induced expression of the *ADT5* transcript (**Figure 1**). The phenotype of *adt5* mutants under FR-BOG conditions mirrored the phenotype of *phyA* and *sig6* (**Figure 4**), which have previously been implicated in light-responsive, phytochrome-dependent plastid development (Alameldin et al., 2020), further highlighting a role for *ADT5* function in light-dependent, photoreceptor-modulated processes. *adt6* mutants exhibit a phenotype where no hormone had any influence on rapid germination of the mutant in darkness (**Figure 2**). Primarily protein expression *in planta* appears to be in the cotyledon in the developing mesophyll (**Supplementary Figure S1**). ADT6 protein may contribute to repressing germination until conditions, in general, are optimal. *ADT6* expression is higher in seeds than *ADT3* and *ADT5* and more similar to *ADT2* (**Figure 1A**) (Hossain et al., 2018), where its action might be in defense or other activities, which may be important in preparing the embryo for germination.

We explored further the relationship between complete darkness and white light as it impacted hypocotyl and root growth (**Figure 5**). The observed responses for *adt6* were distinct from those for *adt5*. The amount of darkness versus light affected hypocotyl and root elongation differently, with both Dc and Wc preventing *adt5* from full (compared to WT) expansion in hypocotyls or roots. 3D4L indicated greater expansion of roots for *adt5*, exceeding WT. Any period of darkness (Dc or 3D4L) promoted greater root expansion of *adt6* compared to WT and 3D4L, promoting longer *adt6* hypocotyls. However, 7 days of white light reduced both shoot and root expansion compared to WT, a trend of reduced root+shoot expansion for all three *adt* mutants. *adt3* responses were unique compared to *adt5* and *adt6*, with longer shoots and roots in complete darkness (7D0L), reduced roots in light (0D7L), and increased shoots in 3D4L. Any darkness increased *adt6* roots (7D0L and 3D4L), and any darkness increased *adt3* shoots (7D0L and 3D4L). ADT proteins may have direct influence on auxin transport or directly affect flavonol concentrations in the root:shoot continuum, or in the meristems in different stages of development (Lewis et al., 2011; Liu et al., 2019). An increased exposure to FR or decreased R:FR impacts auxin transport, and is linked to changes in phenylpropanoid-related compounds, including a reduction in anthocyanins and increased lignin in maize (Afifi and Swanton, 2012). *Adt5* mutants accumulated less anthocyanins in Wc (**Supplementary Figure S4**) in our measurements. Chen et al.’s study of single and multiple ADT gene mutations indicated that ADTs contribute to anthocyanin accumulation, particularly ADT2 at an early stage (Chen et al., 2016).

### 4.2 Roles of ADT1, ADT2, ADT3, and ADT4 in development

ADT1 and ADT4, while not having dramatic phenotypes apparent in this age of plant development, did indicate unique transcript expression profiles (**Figure 1**) that coordinated with the observed expression in the GFP fusions (**Supplementary Figure S1**). ADT2 is important in seed formation and embryo for early survival (Chen et al., 2016). Indeed, both ADT2 and ADT3 (herein and (Para et al., 2016)) indicate impact on earlier processes, with ADT2 being required possibly mostly for seed development (Chen et al., 2016) and defense compounds (Bross et al., 2017) and ADT3 being more important immediately post-germination in development (Para et al., 2016). ADT2 and ADT3 perhaps provide Phe until ADT1, 4, 5, and 6 express in specific tissues of the seedling (photosynthesis in chloroplasts, and roots developing as storage organs). One major event that happens in the seed-to-seedling transition is chloroplast development in the developing leaf cells, where ADT2 and ADT3 may work to coordinate that development, given that different labs have shown that ADT2 (Bross et al., 2017; El-Azaz et al., 2018) and ADT3 (Para et al., 2016) are both required for processes preceding chloroplast development. Phe made via ADTs in general are also recently shown to have a number of specific impacts on photosynthesis and metabolism in older seedlings (~3 weeks old) (Höhner et al., 2018).

### 4.3 Effect of monochromatic light on ADT functions in young seedlings

Based on the results of the growth studies under monochromatic light, the impact of ADT family proteins on Phe pools may contribute to the photoregulation of hypocotyl elongation. Rc, FRc, and Bc light impact hypocotyl elongation of distinct *adt* mutants relative to WT (**Figure 6**). This impact may be due to direct regulation of *ADT* gene expression by light (**Figure 1B**), but could also be due to the general light-associated reduction of Phe pools on growth and development in these mutants. These results suggest an interaction between light and Phe homeostasis. This is further supported by the observed light-impacted differential expression of individual *ADT* genes (**Figure 1**). In some cases, *adt* mutants and phytochrome mutants have similar light-dependent phenotypes. For example, *phyA* and *adt6* have defects in FR- and B-dependent inhibition of hypocotyl elongation responses at 7 days (**Figure 6**) and both *phyB* and *adt6* under Rc were different from WT responses. These data suggest potential phytochrome regulation of the light-associated role for ADT6 and also indicated where *adt6* hypocotyls are reduced in 0D7L, but had greater expansion in 7D0L.

The notable and differential defects of *adt* mutants to hormone treatment in germination assays and defects in light responsiveness suggest interplay between light and hormones in the modulation of Phe homeostasis. Light has been shown to regulate a number of hormonal pathways in plants. The integration of GA signaling and light-dependent phytochrome signaling has been previously shown to involve two PHYTOCHOME-INTERACTING FACTOR (PIF) proteins, PIF3 (Feng et al., 2008) and PIF4 (de Lucas et al., 2008). The transcription factor HY5, which is a key regulator of photomorphogenesis (Oyama et al., 1997), also has been linked to hormonal modulation. HY5 impacts GA (Alabadí et al., 2008), auxin (Cluis et al., 2004; Sibout et al., 2006), and ABA (Chen et al., 2008) signaling. Recent studies have also linked G-box-specific binding transcription factor bZIP16 to light, GA, and ABA signaling through regulating the expression of genes including *PIL5* and *RGL2* (Hsieh et al., 2012). PIL5 (Oh et al., 2006) and RGL2 (Lee et al., 2002) have both been linked to the regulation of seed germination, a phenotype disrupted in *adt* mutants. Light also has a complex regulation of the conversion of ACC to ethylene, dependent on fluence, spectral quality, age of seedlings, and responses to a pulse of light versus continuous irradiations, reviewed in Zdarska et al. (2015). The Rc, FRc, and Bc data of *adt5* or *adt6* compared to WT, as well as the FR-BOG phenotype of *adt5*, indicate that phytochromes and blue light photoreceptors play unique roles with *ADT5* and *ADT6* in development.

### 4.4 Phe and the seedling transition

Development during the seed-to-seedling transition is an intricate multi-component process in higher plants, requiring energy allocation to rapid development and growth, while the emerging seedling also utilizes energy to sense and respond to environmental signals and to avoid and/or defend against potential detrimental environmental factors. Phe is the first committed precursor of the phenylpropanoid pathway, and genetic and biochemical data indicate that Phe is a concentration-limiting substrate (Margna, 1977; Lee et al., 2002; Rohde et al., 2004; Voll et al., 2004; Warpeha et al., 2008). Phe concentrations are likely coordinated with other phenylpropanoid enzymes, several of which have been shown to peak in expression in 3-day-old white light-grown seedlings (Margna et al., 1989a), a developmental age and growth condition utilized in our experiments. Expression of phenylalanine ammonia-lyase (PAL; EC 4.3.1.5), the enzyme catalyzing the first step of the phenylpropanoid pathway that deaminates L-phenylalanine to trans-cinnamic acid, is highly responsive to environmental and developmental cues and induced early in seedling development (Ohl et al., 1989; Kubasek et al., 1992). *pal1 pal2 pal3 pal4* quadruple mutants of Arabidopsis were severely stunted in growth at the early seedling stage and sterile (Lamb et al., 1989). Depending on the *adt* mutant assessed, the growth of seedlings compared to WT was different, so the location of the Phe accumulation in the seedling (tissue) as well as location in cell types may matter. All data considered suggest that through either direct light-mediated signaling impacts on Phe levels or light-associated disruptions in the regulation of Phe synthesis resulting in reduced Phe pools, ADT-dependent Phe homeostasis impacts the coordination of the regulation of the existing energy budget with growth and development. The expression of each *ADT* appears finely tuned to environmental cues and available resources. Perhaps to prepare the seedling to produce the levels of metabolites and structures required, ADTs evolved as mainly type II isoforms with relaxed regulation to enable the massive production of Phe in the seed-to-seedling transition (El-Azaz et al., 2016; El-Azaz et al., 2022). Because of the uniqueness of responses for each ADT, Phe regulation is likely specific to tissues with respect to age and level of development and, hence, may be critical for promoting the successful establishment of seedlings in dynamic environments.

## Data availability statement

The datasets presented in this study can be found in online repositories. The name of the repository and accession number can be found below: NCBI Sequence Read Archive; NCBI Gene Expression Omnibus database: BioProject accession number - PRJNA991474.

## Author contributions

KW and BM designed the experiments. DM, HA, SO, BM, and KW performed experiments. DM, HA, SO, BM, and KW analyzed the data. BM, HA, and KW prepared the article with significant contributions from DM and SO. All authors contributed to the article and approved the submitted version.
