# Supplementary material for: Arogenate dehydratases: unique roles in light-directed development during the seed-to-seedling transition in Arabidopsis thaliana
Source: Front Plant Sci. 2023 Aug 2;14:1220732. doi: 10.3389/fpls.2023.1220732 (PMC10433759; doi:10.3389/fpls.2023.1220732)
Supplement: Supplementary file 1 [file DataSheet_1.pdf]

S1

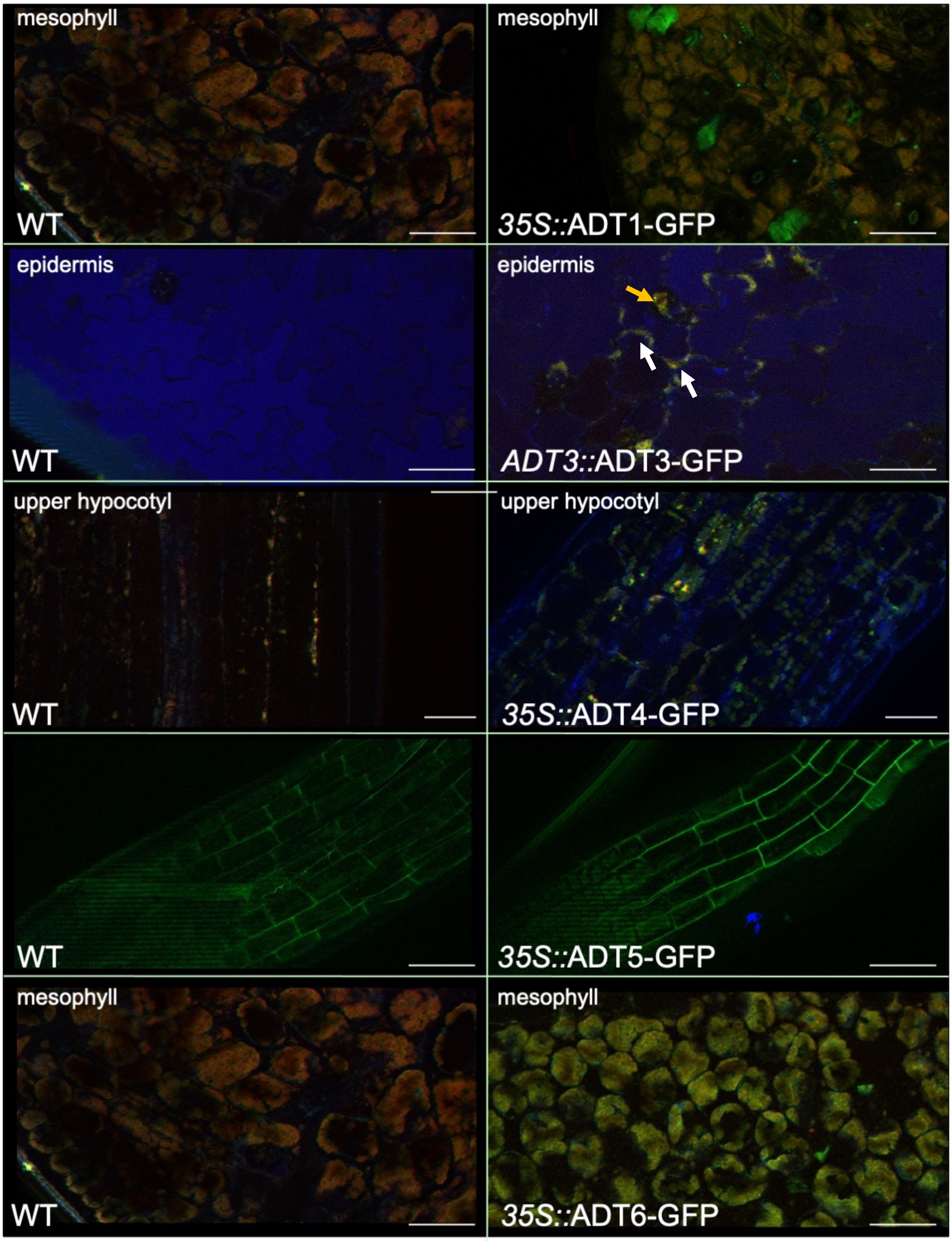


**Supplementary Figure S1. Expression of each ADT in transformed *adt* seedlings.** Null mutant seedlings transformed with *ADT* full length coding region construct (homozygous), indicated on the figure, were grown for 7 d under selection in 16:8 light conditions. On d 7, live seedlings were placed on glass slides, and then were optically sectioned and viewed in their entirety on a deconvoluting microscope with DAPI, FITC, Texas Red LED, then images merged as shown. The merged images allowed us to identify the tissue(s) of expression. Shown are representative 1.0 µm slices parallel to the long axis of the intact seedling or cotyledon. There were no ADT members highly expressed in all tissues. Expression was tissue-specific. Arrows indicate specific expression (ADT3, orange arrow developing guard cell, white arrows plasma membrane). Images are representative of the data. Scale bar length=50 µm.

S2

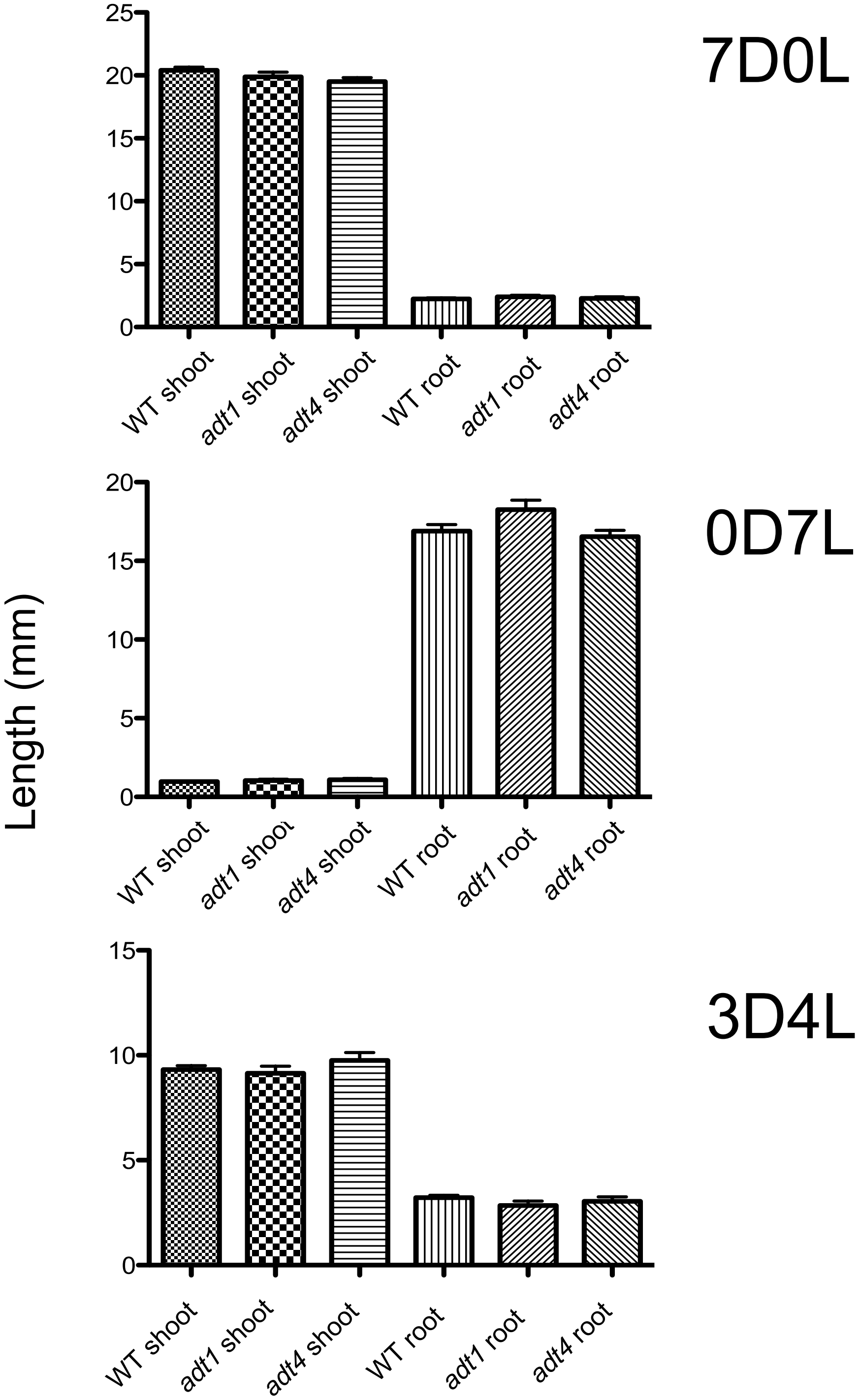


**Supplementary Figure S2. Shoot and root lengths of *adt1* and *adt4* indicated no difference compared to WT.** Wild-type and *adt* seedlings were grown vertically on sucrose-free 0.5XMS plates under same conditions as Fig. 5. Growth conditions are grouped as (a) continuous darkness (7D0L), (b) continuous light (0D7L), or (c) a dark-light transition (3 days darkness; 4 days light, 3D4L). Data were entered in to GraphPad Prism to make bar graphs and determine SD. Statistics as described in Methods, and involve a non-parametric *t* test applying Kolmogorov-Smirnov, performed in the same way as for Fig. 5 data, described in Methods.

S3

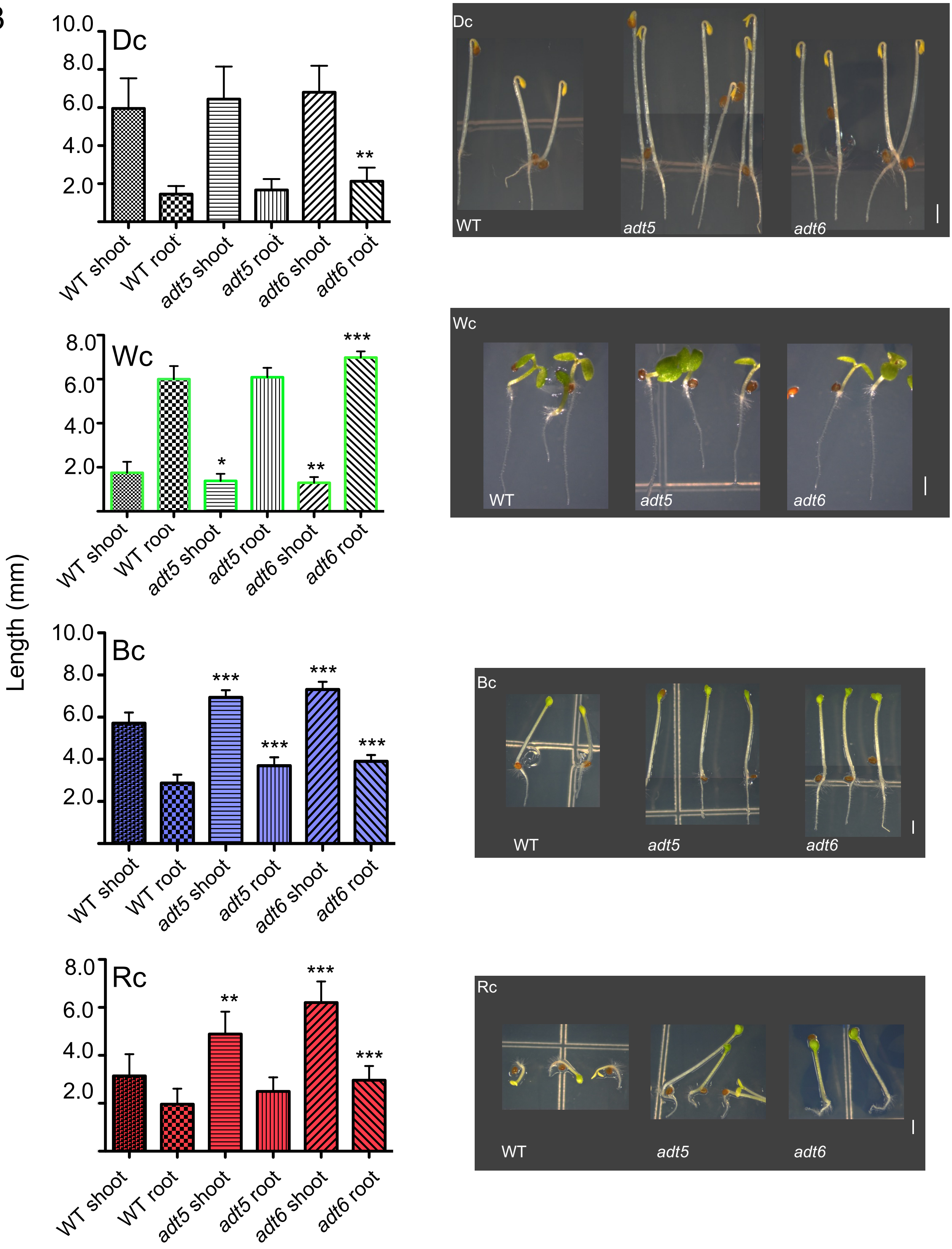


**Supplementary Figure S3. Response of *adt5* and *adt6* to low fluence light as 3-day seedlings.** Wild-type and *adt5* and *adt6* seedlings were grown vertically on sucrose-free 0.5XMS plates as described for Fig. S1, but were maintained under Dc, Wc, Bc or Rc as described in Methods. Seedlings were imaged and shoots and roots measured at 72 h after vernalization (3 days), where data were entered in to GraphPad Prism to make bar graphs and determine SD. Statistics as described in Methods, and involve a non-parametric t test applying Kolmogorov-Smirnov. Size bar = 1.0mm. Images are representative.

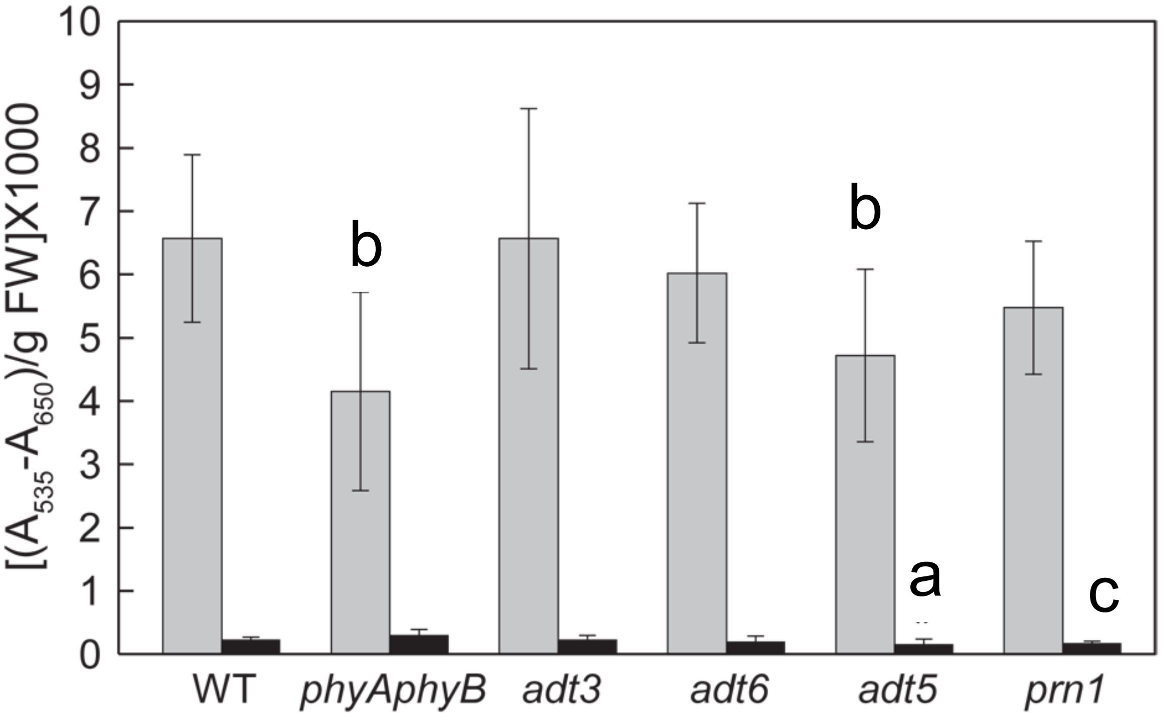


**Supplementary Figure S4. Anthocyanin content of wild-type, *adt* and *prn1* seedlings.** Seedlings were grown at 22° C on Phytablend medium containing 1% sucrose for 5 d under darkness or Wc illumination of 100 µmol $m^{-2}$ $s^{-1}$. *phyAphyB* mutant seedlings are included as a control. Bars, gray bars (Wc) and black bars (darkness), represent the mean (± SD) of four independent measurements. Statistical analysis was conducted using two-tailed, unpaired Mann-Whitney U-test to compare the means of anthocyanin contents per mg of fresh weight for a particular mutant line relative to WT. Letters above bars: a, $p < 0.001$; b, $p < 0.01$; c, $p \leq 0.05$.
